# *In Vivo* Visualization of Active Polysynaptic Circuits With Longitudinal Manganese-Enhanced MRI (MEMRI)

**DOI:** 10.3389/fncir.2018.00042

**Published:** 2018-05-22

**Authors:** Suellen Almeida-Corrêa, Michael Czisch, Carsten T. Wotjak

**Affiliations:** ^1^Department of Stress Neurobiology & Neurogenetics, Max Planck Institute of Psychiatry, Munich, Germany; ^2^Core Unit Neuroimaging, Max Planck Institute of Psychiatry, Munich, Germany

**Keywords:** manganese-enhanced MRI, neuroimaging, brain connectomics, Mn^2+^ transport, barrel-cortex, whiskers, sensory deprivation

## Abstract

Manganese-enhanced magnetic resonance imaging (MEMRI) is a powerful tool for *in vivo* non-invasive whole-brain mapping of neuronal activity. Mn^2+^ enters active neurons via voltage-gated calcium channels and increases local contrast in T_1_-weighted images. Given the property of Mn^2+^ of axonal transport, this technique can also be used for tract tracing after local administration of the contrast agent. However, MEMRI is still not widely employed in basic research due to the lack of a complete description of the Mn^2+^ dynamics in the brain. Here, we sought to investigate how the activity state of neurons modulates interneuronal Mn^2+^ transport. To this end, we injected mice with low dose MnCl_2_ 2. (i.p., 20 mg/kg; repeatedly for 8 days) followed by two MEMRI scans at an interval of 1 week without further MnCl_2_ injections. We assessed changes in T_1_ contrast intensity before (scan 1) and after (scan 2) partial sensory deprivation (unilateral whisker trimming), while keeping the animals in a sensory enriched environment. After correcting for the general decay in Mn^2+^ content, whole brain analysis revealed a single cluster with higher signal in scan 1 compared to scan 2: the left barrel cortex corresponding to the right untrimmed whiskers. In the inverse contrast (scan 2 > scan 1), a number of brain structures, including many efferents of the left barrel cortex were observed. These results suggest that continuous neuronal activity elicited by ongoing sensory stimulation accelerates Mn^2+^ transport from the uptake site to its projection terminals, while the blockage of sensory-input and the resulting decrease in neuronal activity attenuates Mn^2+^ transport. The description of this critical property of Mn^2+^ dynamics in the brain allows a better understanding of MEMRI functional mechanisms, which will lead to more carefully designed experiments and clearer interpretation of the results.

## Introduction

The dissection of neuronal pathways involved in specific brain networks underlying distinct behavioral outputs is of outmost interest to modern neuroscience. Currently employed methods in basic research include local brain injections of neuronal anterograde (Gerfen and Sawchenko, 1984; Veenman et al., 1992) and retrograde tracers (Arvidson, 1977; Schmued and Fallon, 1986; Quattrochi et al., 1989) or viral vectors (Wickersham et al., 2007; Zeng et al., 2017; Zingg et al., 2017). They rely on the investigation of the pathways *post-mortem*, via histological analysis of brain slices or cleared brains when employing CLARITY based protocols for example (Chung et al., 2013). Moreover, these methods are limited to pre-defined regions of interest, given that they require targeted brain injections. A lot has been learned about neuronal circuits employing these tools, however, a non-invasive technique that would allow follow-up investigations comparing the same animals overtime is still desirable. Here, we focus on manganese-enhanced magnetic resonance imaging (MEMRI) as a powerful alternative.

MEMRI has the potential to non-invasively map whole-brain activity and identify structures related to a specific task (Chen et al., 2007, 2013; Bissig and Berkowitz, 2009; Eschenko et al., 2010; Bangasser et al., 2013; Hoch et al., 2013; Tang et al., 2016; Laine et al., 2017) since Mn^2+^ enters active neurons through voltage-gated calcium channels (Drapeau and Nachshen, 1984) (e.g., Ca_v_1.2; Bedenk et al., 2018), and is transiently kept intracellularly (Gavin et al., 1990). Mn^2+^ shortens the T_1_ relaxation time of water (Spiller et al., 1988; Nordhøy et al., 2004) leading to a contrast increase in T_1_-weigthed images (Pautler and Koretsky, 2002). Brain structures that accumulate Mn^2+^ can be detected as hotspots in T_1_-weighted images, indicating higher neuronal activity in these areas (Lin and Koretsky, 1997). This technique modality is also referred to as activation-induced manganese-dependent MRI (AIM-MRI) (Tambalo et al., 2009). If the integrity of the blood-brain barrier is disrupted, even dynamic accumulation of Mn^2+^ can be observed in a single experimental session (DAIM-MRI) (Aoki et al., 2002).

MEMRI is also used for tract-tracing (for review see Pautler, 2004), since Mn^2+^ can be axonally transported to neuronal terminals after local MnCl_2_ administration (Sloot and Gramsbergen, 1994; Pautler et al., 1998), revealing the underlying circuitry of the injection target. During this process, Mn^2+^ may cross one or more synapses (Pautler et al., 1998).

We have recently shown that Mn^2+^ preferentially accumulates in projection terminals of the active entrance sites after systemic MnCl_2_ administration (Bedenk et al., 2018). This feature of Mn^2+^ allows for the combination of activity-induced dissection of structures related to a specific behavior, and the connectomics analysis of the neuronal pathways underlying these brain structures. In that way, MEMRI does not only provide a snapshot of the structures active in response to a given task, but also reveals the downstream connectivity of these brain structures. This results in a functional connectivity map. Furthermore, the possibility of scanning the same animals at different time points allows for dynamic investigations of the functional circuitry in a within-subject fashion, thus reducing the number of required subjects while increasing the power of such studies (3-Rs principle for ethical use of animals in testing).

Despite those features, MEMRI is still not widely used, partially due to toxic side effects, but also due to insufficient information regarding Mn^2+^ dynamics in the brain, confounding the interpretation of the results. Some properties, such as activity-dependent entrance into cells via voltage-gated calcium channels (Drapeau and Nachshen, 1984), transient intracellular storage (Gavin et al., 1990), and preferential accumulation in projection terminals (Bedenk et al., 2018) have previously been reported. However, other properties such as the influence of neuronal activity state on intracellular Mn^2+^ storage and axonal transport have been debated in the literature with inconclusive findings. Therefore, a complete description of Mn^2+^ dynamics in the brain is still lacking.

To address this, we conducted a longitudinal within-subject study to investigate whether, following systemic injections of MnCl_2_, the transport of Mn^2+^ is dependent on neuronal activity elicited by sensory stimulation. As a model pathway for this study we chose the whiskers-barrel cortex system, based on its well-described and defined connectivity (for examples see Chmielowska et al., 1989; Aronoff et al., 2010; Zakiewicz et al., 2014) and the property of sensory stimulation by whisking resulting in a strong and specific increase on neuronal activity at the corresponding contralateral barrel cortex (Woolsey and Van der Loos, 1970; Axelrad et al., 1976; Peron et al., 2015). As such, we aimed to compare the contrast patterns observed with MEMRI (i) following systemic MnCl_2_ injections in mice with intact whiskers in enriched sensory housing conditions, and (ii) after the same mice were partially sensory deprived (unilateral whisker trimming). We hypothesized that ongoing sensory input would lead to accelerated clearance of Mn^2+^ in the corresponding barrel cortex with a concomitant relative increase in Mn^2+^ accumulation in efferent structures.

## Materials and methods

All experiments were carried out according to the European Community Council Directive 2010/63/EEC. All experimental procedures were approved by the local government of Upper Bavaria (AZ 142-12). Every effort was done to keep the number of experimental subjects at a minimum and to avoid animal suffering.

### Animals

Adult male C57BL/6N mice (*n* = 9) from our local breeding stock (Max Planck Institute of Biochemistry, Martinsried, Germany) were kept in groups of 3 per cage with food and water *ad libitum*, under a 12 h dark/light inverted cycle (lights on at 07h30), in a room with controlled temperature and humidity. After transfer to the local animal facility at the Max Planck Institute of Psychiatry, mice were allowed to get accustomed to the holding conditions (standard macrolon cages type II; 267 × 207 × 140 mm, floor area 370 cm^2^; Tecniplast, Italy) for at least 10 days before experiments started. Mice were 3 to 4 months old at the time of experiments. Intraperitoneal injections described next were conducted between 16h00 and 20h00.

### Drugs

- MnCl_2_ × 4H_2_O (Sigma-Aldrich, Steinheim, Germany) was dissolved in 0.9% NaCl to a final concentration of 50 mM (4947.5 mg−500 mL saline). The pH was adjusted to 6.95 with HCl and NaOH.
- Ketamine + xylazine solution: 138 mg of ketamine and 6.8 mg of xylazine/10 mL solution (0.9% NaCl).

### Experimental procedures

Mice (3/cage) were housed in large type III cages (425 × 266 × 155 mm, floor area 820 cm^2^; Tecniplast, Italy) enriched with extra nesting material, plastic hair curlers of two different sizes (2 big, 36 mm radius; 3 medium, 36 mm radius), used as texturized tunnels (textures on the inner and outer part), and a hanging thread at the metal lid with a another small hair curler/tunnel (28 mm radius). Mice were kept in the same group under this 8. condition for 8 days, until scanned (scan 1), followed by another 7 days of enriched housing and a second scan (scan 2).

All mice received intraperitoneal injections of 20 mg/kg MnCl_2_ (Sigma-Aldrich, Steinheim, Germany) every 24 h for eight consecutive days (8 x 20/24 h), in order to minimize physiological side effects (adapted from Grünecker et al., 2010; Bedenk et al., 2018). Mice were always weighted immediately before injections to monitor animal's health status and to guarantee the correct dose would be injected every day.

On day 8, animals (3 per day) were individually anesthetized with a mixture ketamine and xylazine (i.p., injection of 0.1 mL/10 g mice) and transferred to the MRI room. With ketamine we aimed to block NMDA receptors (Anis et al., 1983) and thus, to avoid further Mn^2+^ neuronal entrance (Itoh et al., 2008) during the transport of the animals between rooms. For the scanning procedure, see below.

Immediately after scan 1, and still under sedation, animals had all their whiskers trimmed close to the skin on the left side of the snout. The right side was untouched. After trimming, animals were put back in the enriched cages. The trimming procedure was repeated every 2 days (under light isoflurane anesthesia) to avoid re-growth of the whiskers. After scan 1, animals received no further MnCl_2_ injections.

**Figure 1:**
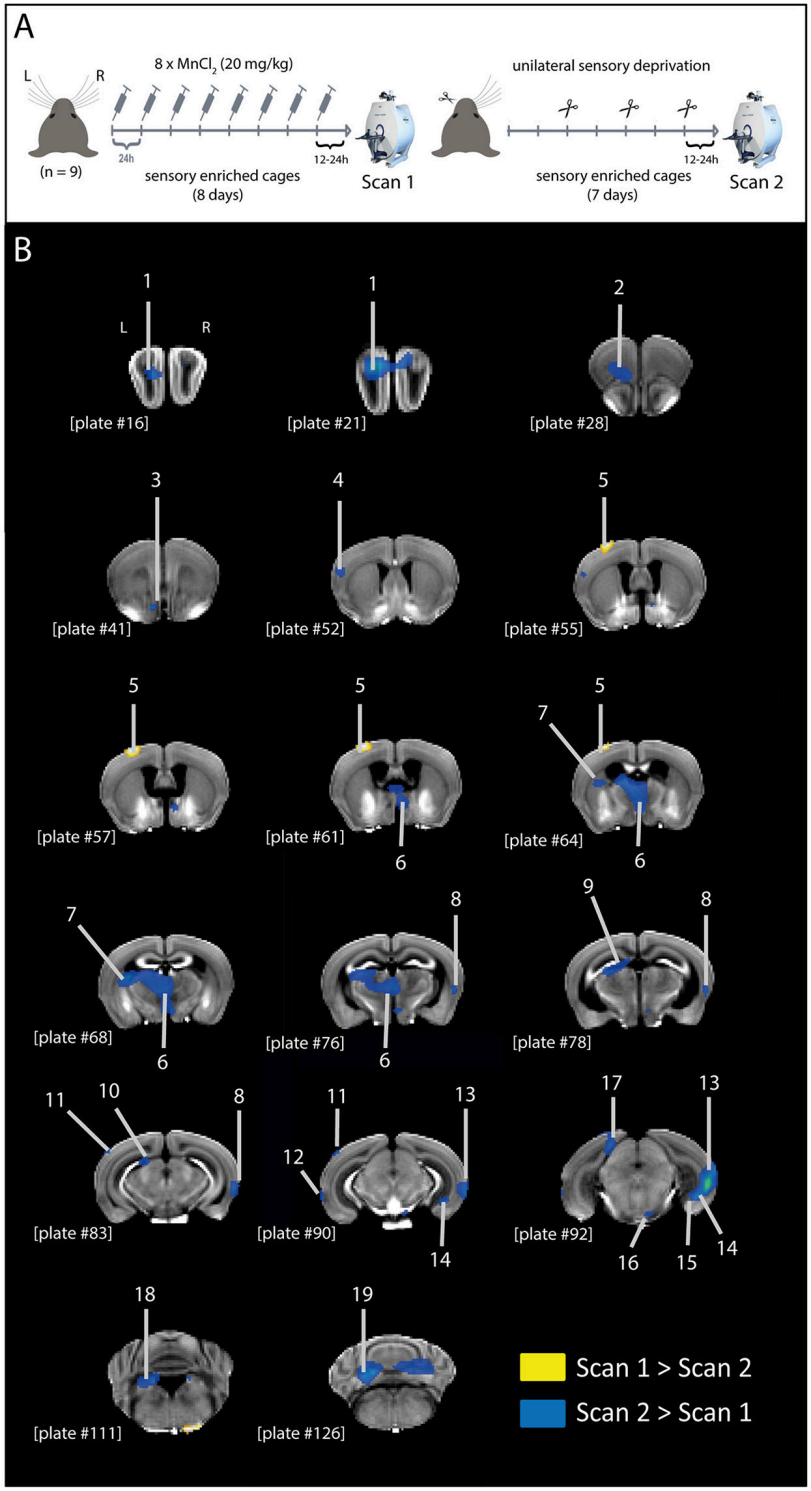
MEMRI contrast differences after unilateral sensory deprivation. **(A)** Graphic representation of experimental design. Mice were treated with MnCl_2_ (20 mg/kg; i.p.) for 8 days, while housed in a sensory enriched environment, until scan 1. Immediately after scan 1, mice had their left whiskers trimmed (procedure repeated every 2 days) and remained in the sensory enriched environment for 7 more days without further MnCl_2_ injections, until scan 2. **(B)** Representative coronal brain slices indicating the structures showing differential MEMRI signal in scans 1 and 2 (yellow: scan 1 > scan 2; blue: scan 2 > scan 1). Brain structures indicated in the figure: 1, olfactory bulb; 2, orbital area; 3, islands of Calleja; 4, supplemental somatosensory area; 5, barrel cortex; 6, medial thalamic nuclei; 7, caudoputamen; 8, temporal association area; 9, anterior pretectal nucleus; 10, nucleus of the optic tract; 11, anterolateral visual area; 12, perirhinal area; 13, temporal association area + ectorhinal area + perirhinal area; 14, subiculum—ventral part; 15, dentate gyrus—ventral part; 16, pontine nuclei; 17, retrosplenial area; 18, superior vestibular nucleus; 19, cerebellum. Plate numbers under brain slices correspond to the reference plate of the Allen Mouse Brain Atlas used to define the structures.

On the last day of enrichment after scan 1, animals (3 per day) were again individually anesthetized with a mixture of ketamine and xylazine (i.p., injection of 0.1 mL/10 g mice) and transferred to the MRI room for scan 2. For graphic representation of the experimental design see Figure 1A.

### Manganese-enhanced magnetic resonance imaging (MEMRI)

Twelve to twenty-four hours after the last of 8 daily MnCl_2_ injections, a first MRI scanning took place (scan 1). Seven days after scan 1, the second MRI scanning took place (scan 2).

All MEMRI experiments were conducted on a 7T Avance Biospec 70/30 scanner (Bruker BioSpin, Ettlingen, Germany). In brief, essentially as described before (Bedenk et al., 2018), mice were fixed in supine position on a saddle-shaped receive-only coil. Head fixation was achieved using a stereotactic device and the frontal teeth were fixed with a surgical fiber. Once fixed in the coil, mice were kept anesthetized with an isoflurane-oxygen mixture (1.0–1.5 vol %, with an oxygen flow of 1.2–1.4 L/min) (Delta Select, Germany). A rectal thermometer was used for body temperature monitoring (Thermalert TH-5, Physitemp Instruments, USA). Body temperature was kept between 36.5 and 37.5°C using a water-based heating pad. Pulse rate was continuously monitored by a plethysmographic pulse oxymeter (Nonin 8600V, Nonin Medical Inc., USA).

T_1_-weighted (T1w) brain images were acquired using a 3D gradient echo pulse sequence [TR = 50 ms, TE = 3.2 ms, matrix size = 128 × 106 × 106 zero filled to 128 × 128 × 128, field of view (FOV) = 16 × 16 × 18 mm^3^, number of averages = 10, resulting in a spatial resolution of 125 × 125 × 140.6 μm^3^].

### MRI data post-processing

Images were reconstructed in Paravision (Bruker, BioSpin, Ettlingen, Germany) and transferred to standard ANALYSE format. Further post-processing was performed using SPM 8 (www.fil.ion.ucl.ac.uk/spm). T1w-images were bias-corrected using the algorithm implemented in SPM8, minimizing the entropy of the image histogram. In this way we could remove intensity gradients introduced by differences in the distance between surface receiver coil and the brain structures (Milchenko et al., 2006). For each individual subject the brain was then extracted using the RATS software (https://www.estima.com/ratsmain.shtml). Images were spatially normalized in two steps: In the first step, we generated a study-specific group template. For this purpose, we initially normalized all individual brain extracted images to a representative single subject image of good quality. The study-specific template image was then calculated as the mean image of all normalized images of this first step. In a second normalization step, this study-specific template was then used as the new target image for normalization. Doing so, we aimed at minimizing individual regional discrepancies in the final normalized images (Huang et al., 2010). Finally, all normalized images were smoothed with a Gaussian kernel of eight-times the image resolution (1.0 × 1.0 × 1.124 mm^3^ at full-width half maximum). Data were analyzed using a paired *t*-test (scan 1 and scan 2), along with cerebrospinal fluid (CSF) intensities as a nuisance regressor. To account for unspecific global intensity changes due to Mn^2+^ wash-out between the two measurements (Grünecker et al., 2013), global image intensities were added as another nuisance regressor. Calculation of the global mean regressor was automatically performed in the generation of the ANCOVA model in SPM8: the global mean is calculated as the mean intensity of all voxel inside the standard analysis mask. By default, this mask includes all voxels which show an intensity larger than 1/8^*^(mean of all image voxels).

### Definition of brain structures

All the brain structures shown in Figure 1 and listed in Table 1 were defined using the Allen Mouse Brain Atlas (Lein et al., 2006) (http://mouse.brain-map.org/experiment/thumbnails/100048576?image_type=atlas) as a reference. The only exception is the “islands of Calleja,” defined based on “10. The Mouse Brain in Stereotaxic Coordinates” (Franklin and Paxinos, 2007).

**Table 1:** List of structures showing differential MEMRI signal between scans 1 and 2, ipsi or contralateral to the reference point (left barrel cortex).

		**MEMRI signal** ≠		Barrel cortex efferent?^*^	
**Brain structures**	**# on Figure 1**	**ipsi (L)**	**contra (R)**	**ipsi (L)**	**contra (R)**
Olfactory bulb—anterior	1				
Olfactory bulb—posterior	1				
Orbital area	2				
Islands of Calleja (striatum)	3				
Supplemental somatosensory area	4				
**BARREL CORTEX**	**5**				
Medial thalamic nuclei—anterior	6				
Medial thalamic nuclei—posterior	6				
Caudoputamen	7				
Temporal association areas	8				
Lateral posterior nucleus of the thalamus	6				
Dorsal part of the lateral geniculate complex	6				
Parafascicular nucleus	6				
Posterior complex of the thalamus	6				
Anterior pretectal nucleus	9				
Lateral posterior nucleus of the thalamus	6				
Nucleus of the optic tract	10				
Anterolateral visual area	11				
Perirhinal area	12				
Ectorhinal area	13				
Subiculum—ventral part	14				
Dentate gyrus—ventral part	15				
Pontine nuclei	16				
Retrosplenial area	17				
Superior vestibular nucleus (medulla)	18				
Cerebellum	19				

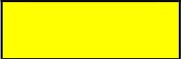
 Scan 1 > Scan 2 
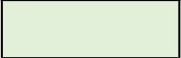
 1st order efferents

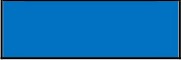
 Scan 2 > Scan 1 
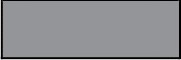
 2nd order efferents

* *based on the following references: White and DeAmicis, 1977; Ohara et al., 1980; Montero and Scott, 1981; Ohara and Lieberman, 1981, 1985; Hoogland et al., 1987, 1991; Cornwall and Phillipson, 1988; Welker et al., 1988; Chen et al., 1992; Raos and Bentivoglio, 1993; Bourassa et al., 1995; Hazrati et al., 1995; Pinault et al., 1995; Pinault and Deschenes, 1998; Veinante et al., 2000; Wright et al., 2000; Wang et al., 2005; Aronoff et al., 2010; Zakiewicz et al., 2014; Tang et al., 2016; Guo et al., 2017; Sumser et al., 2017*.

### Statistics and data presentation

We interrogated the contrast scan 1 > scan 2 using a strict family-wise error corrected threshold of p_FWE,cluster_ < 0.05, with a collection threshold of p_uncorrected_ < 0.001 (Woo et al., 2014), which is in accordance with other MEMRI studies (Lutkenhoff et al., 2012; Laine et al., 2017). Due to expected dilution of Mn^2+^ concentrations after cessation of the MnCl2 injections, relative local increases of Mn^2+^ accumulation in the second scan (scan 2 > scan 1) were only assessed qualitatively at an uncorrected threshold of *p* < 0.05 (cluster extent 20).

Voxel-wise analysis of the MR images was performed in SPM8 (www.fil.ion.ucl.ac.uk/spm). Graphics of activation maps have been created in MRICro (www.cabiatl.com/mricro). All images were ultimately arranged in Adobe Illustrator 10.0.3 (Adobe Systems Inc., NY, USA).

## Results

After correcting for the unspecific global decrease of Mn^2+^ between the experimental time points, we identified only a single cluster showing higher Mn^2+^ intensity in the first scan compared to the second (scan 1 > scan 2). This cluster was located in the left barrel cortex (p_FWE,cluster_ = 0.009, cluster extent 236 voxel), representing activity of the untrimmed whiskers (Figure 1B; Table 1).

In the inverse contrast (scan 2 > scan 1), a number of brain structures could be detected to show a stronger intensity at time point 2 (Figure 1B; Table 1), mainly located in the left hemisphere.

The higher signal in the left barrel cortex (corresponding to the untrimmed whiskers) in scan 1 compared to scan 2, and the lack of difference in the right barrel cortex (corresponding to the trimmed whiskers) suggests that the sensory blockage by whisker trimming attenuated the Mn^2+^ transport to projection terminals. This hypothesis is further supported by the clusters showing higher Mn^2+^ intensity in the second measurement compared to the first (scan 2 > scan 1), which include a large number (85% of total) of efferents of the left barrel cortex (Table 1). Therefore, we conclude that Mn^2+^ is transported from the uptake site to its projection terminals, in an activity-dependent manner.

## Discussion

**Figure 2:**
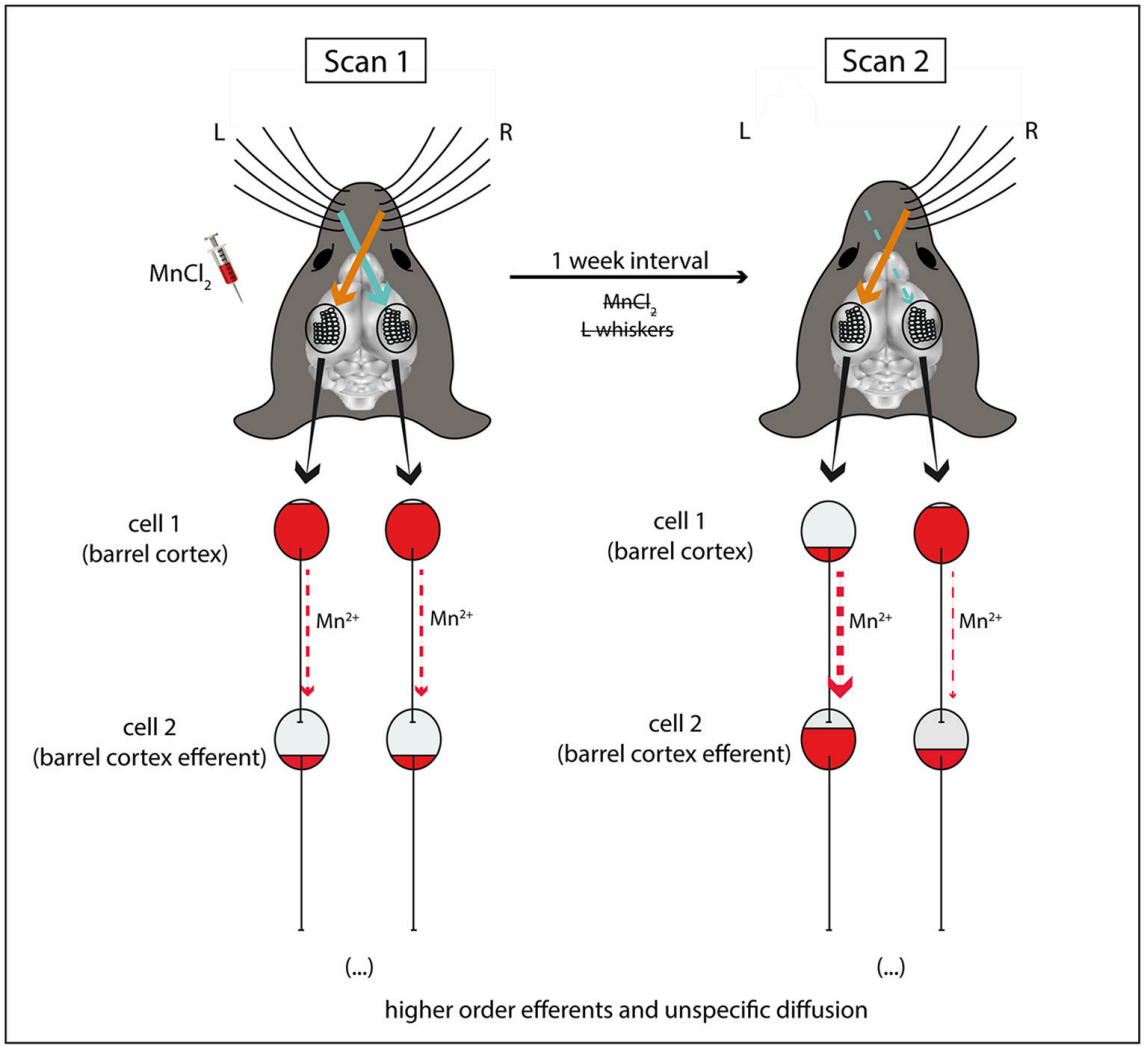
Schematic explanation for the differences in Mn^2+^ accumulation observed after unilateral sensory deprivation. In the end of sensory enriched housing with intact whiskers and repeated MnCl_2_ injections, MEMRI (scan 1) reveals equal bilateral accumulation of Mn^2+^ in the barrel cortices. 1 week later (scan 2) after unilateral sensory deprivation (left side), Mn^2+^ is cleared from the left but not right barrel cortex (cell 1), due to ongoing sensory inputs from the intact whiskers of the contralateral right side. At the same time, Mn^2+^ is accumulated in efferent brain structures downstream to the left barrel cortex (cell 2) following activity-dependent axonal/transsynaptic transport. For simplicity reasons, this scheme does not depict the afferences from brain stem structures and the thalamus which relay sensory information from the whiskers to the barrel cortex.

Here we show that, after systemic MnCl_2_ injections, both intra- and interneuronal transport of Mn^2+^ is accelerated by the continuous activity of the afferent cells in the brain, when compared to a unilaterally sensory deprived pathway. This conclusion was based on the following observations: (i) only the barrel cortex of the corresponding untrimmed whiskers showed higher MEMRI signal in scan 1 compared to scan 2; (ii) most of the structures that showed higher MEMRI signal in scan 2 compared to scan 1 are efferent to the barrel cortex (Table 1; Figure 2).

### The whisker-barrel cortex system

The mouse barrel-cortex system was chosen as a model due to its well characterized connectivity (Woolsey and Van der Loos, 1970; Welker, 1976) and because of its property of strong and defined neuronal activation in the barrel cortex contralateral to its specific sensory input (Woolsey and Van der Loos, 1970; Axelrad et al., 1976; Peron et al., 2015). Therefore, it is the perfect model system to study changes in neuronal activity due to sensory stimulation or deprivation, and the underlying Mn^2+^ dynamics related to neuronal activation. This pathway was already used elsewhere to map tactile sense-evoked activity with MEMRI (Weng et al., 2007), BOLD (Lu et al., 2004; de Celis Alonso et al., 2012) and CBV fMRI (Lu et al., 2004) after mechanical whisker stimulation in rats; also with MEMRI after blood-brain barrier ultrasonic disruption and mechanical whisker stimulation in mice (Howles et al., 2010).

For the sake of simplicity, in our conclusion scheme (Figure 2) we represented neurons of the barrel cortex as cell 1 (reference point). These neurons are the cortical representation of a mouse's contralateral whiskers (Woolsey and Van der Loos, 1970; Ferezou et al., 2006). However, it is worth highlighting that after stimulation of the whiskers, sensory information is initially processed by the trigeminal nuclei of the brainstem, followed, in parallel, by the ventroposterior medial (VPM)—lemniscal and extralemniscal pathways—and the posterior medial (POm) nuclei of the thalamus—paralemniscal pathway—before reaching the barrel cortex (for review Petersen, 2007; Diamond et al., 2008). Therefore, the barrel cortex cells are already downstream to other brain structures which may take up Mn^2+^ in an activity-dependent manner.

Most of the brain structures which showed higher Mn^2+^ levels in scan 2 compared to scan 1 turned out to be efferent to the left barrel cortex, including monosynaptic (1st order) and polysynaptic (2nd order) projection sites (Table 1). This connectivity analysis and assignment was made taking the vast barrel cortex connectivity data in the literature into account (for examples see Aronoff et al., 2010; Zakiewicz et al., 2014). Second order efferents were here defined as the projections from either of the two main outputs of the barrel cortex, namely: the thalamic reticular nucleus and the posterior complex of the thalamus (Hoogland et al., 1987; Wright et al., 2000). Some brain structures, especially medial thalamic nuclei, are both 1st and 2nd order efferents. In these cases, they were just assigned as 1st order efferents.

An important point to mention is that most of the whisker-barrel connectivity literature is based on rat experiments. Large part of this data (Ohara et al., 1980; Montero and Scott, 1981; Ohara and Lieberman, 1981, 1985; Cornwall and Phillipson, 1988; Chen et al., 1992; Raos and Bentivoglio, 1993; Bourassa et al., 1995; Hazrati et al., 1995; Pinault et al., 1995; Pinault and Deschenes, 1998; Veinante et al., 2000; Wright et al., 2000; Wang et al., 2005; Zakiewicz et al., 2014) was included in our analysis to assign structures as barrel cortex efferents. Given the similarities of the rat and mouse nervous system, we do not believe that inter-species differences could falsely or significantly impact the connectivity analysis presented here. However, we acknowledge that small differences between the barrel system connectivity of these species were already reported (Kichula and Huntley, 2008).

The results shown here included most, but not all the barrel cortex efferents previously described. In fact, some of its main outputs, such as the motor cortex or the thalamic reticular nucleus, did not show a differential MEMRI signal in scan 2 compared to scan 1. This might be ascribed to the fact that Mn^2+^ can be transsynapticaly transported, as already reported elsewhere (Saleem et al., 2002; Pautler et al., 2003; Murayama et al., 2006; Bearer et al., 2007), and also shown here by the geniculate and parafascicular nuclei of the thalamus, which represent 2nd order efferents from the barrel cortex and 1st order efferents from the reticular nucleus. This might lead to an additional dilution of the Mn^2+^ contrast, hindering its detection by voxel wise brain analysis. Another possibility is that the MEMRI signal might have been filtered out in large structures which receive diffused rather than focused projections, such as the motor cortex, because the signal intensity tends to be higher in compact and densely connected structures (Aoki et al., 2004; Bedenk et al., 2018).

It is important to note that our protocol of partial sensory deprivation (whisker trimming) was applied to adult mice only (3–4 months old) and did not include follicle removal or cauterization. Moreover, considering the short duration of the deprivation (7 days), we do not believe that the results of this study are a consequence of cortical map plasticity, widely described in neurodevelopmental and plasticity studies using the barrel-cortex system model (Van der Loos and Woolsey, 1973; Woolsey and Wann, 1976; Levin and Dunn-Meynell, 1991; Dunn-Meynell et al., 1992; Siucinska and Kossut, 1994; Melzer and Smith, 1996; Kossut and Juliano, 1999; Fox, 2002; Allen et al., 2003; Rema et al., 2003; Schierloh et al., 2003; Shepherd et al., 2003; Dubroff et al., 2005; Fox and Wong, 2005; Shoykhet et al., 2005; Frostig, 2006; Lee et al., 2007; Schubert et al., 2007; Drew and Feldman, 2009; Wu et al., 2011; Gainey et al., 2016; Jacob et al., 2017).

### Mn^2+^ administration, toxicity, and decay

MEMRI studies have already employed different routes for Mn^2+^ administration, such as intracerebral injections (Pautler et al., 2003; Watanabe et al., 2004; Yang et al., 2011), intranasal aerosols (Henriksson et al., 1999; Pautler and Koretsky, 2002; Lehallier et al., 2012), intravitreal injection (Pautler et al., 1998; Bearer et al., 2007; Luo et al., 2012), and topic eye application (Lin et al., 2014). These methods are however invasive and often toxic (Bearer et al., 2007; Luo et al., 2012; Lin et al., 2014). Systemic injections have a reduced risk of toxicity if fractionated (Grünecker et al., 2010), or continuously delivered with osmotic mini pumps (Sepulveda et al., 2012; Poole et al., 2017). The delayed and limited diffusion of Mn^2+^ to the brain should also be considered. In each case, care must be taken to find an optimal balance between a sufficient dose to reach the best contrast while minimizing the potential side/toxic effects of Mn^2+^ in the brain. The use of systemic methods for delivering of MnCl_2_ has clear advantages, e.g., in case of prolonged behavioral procedures. In some cases, however, systemic treatment has to be combined with the disruption of the blood-brain barrier (BBB), e.g., by mannitol injection (Lin and Koretsky, 1997; Aoki et al., 2002) or by ultrasound (Howles et al., 2010), in order to allow the Mn^2+^ to quickly reach the brain. In these cases, a single MnCl_2_ spike injection can be applied. Even with the use of relatively small doses for a single shot that did not cause major apparent side/toxic effects, small impairments as transient motor deficit in skilled reaching, rears, and activity was already described in rats (Alaverdashvili et al., 2017). This limitation should be considered, especially when designing studies with behavioral experiments where fine motor skills are necessary. For longer term investigations (from many hours to days) the disruption of the BBB is not necessary (Yu et al., 2005; Kuo et al., 2006), given that Mn^2+^ can reach the brain and accumulate in a activity-dependent manner in the structures related to the challenge/task performed at least few hours before. This applies in particular to the paradigm used here, where we “pre-loaded” the cells with Mn^2+^ before the experimental intervention (whiskers trimming). Our data suggest this procedure might also be used for acute behavioral challenges where mice could be first treated with MnCl_2_ to reach sufficient contrast, followed by repeated scanning before and after the challenge.

One should also not overlook clearance of Mn^2+^in the brain when scans are performed long (more than 24 h) after the MnCl_2_ injections have stopped. We previously reported that the half-life of Mn^2+^, after an 8 × 30 mg/kg MnCl_2_ injection protocol, is about 5–7 days, depending on the brain structures (Grünecker et al., 2013). This point was taken into consideration in our analysis comparing scans 1 and 2, which were performed 1 week apart.

### The interplay of neuronal activity and Mn^2+^ axonal and transsynaptic transport

Previous studies already investigated the possible role of neuronal activity in Mn^2+^ axonal and transsynaptic transport in specific pathways with different protocols and obtained, somewhat, contradicting results. For instance, it was shown that Mn^2+^ is co-released with neurotransmitters after stimulation with high K^+^ (Takeda et al., 1998), indicating that Mn^2+^ transport is dynamically linked to neural signaling. Later, many groups mapped sensory system activation in response to specific odors (Pautler et al., 1998; Pautler and Koretsky, 2002; Chuang et al., 2009; Lehallier et al., 2012), visual (Bissig and Berkowitz, 2009), or acoustic stimulation (Yu et al., 2005), supporting the idea that Mn^2+^ transport is activity-dependent. One of these studies (Bearer et al., 2007) employed transgenic blind mice to investigate activity-dependency in Mn^2+^ dynamics after intravitreal MnCl_2_ injection and concluded that “Mn^2+^ is not transmitted efficiently across synapses in the absence of electrical activity in this system,” whereas uptake and axonal transport remained intact. This last conclusion is supported by the results of Lowe et al. (2008) showing no difference in MEMRI signal intensity in the visual system between groups treated with MnCl_2_ only or in combination with cell activity blockers (APB or TTX). On the other hand, accelerated Mn^2+^ transport after MnCl_2_ co-treatment with AMPA was already described (Wang et al., 2015), indicating that axonal transport of Mn^2+^ is dynamically modulated by neuronal activity. In fact, pharmacological blockage of calcium channels also blocked this accelerated transport (Wang et al., 2015). Using the song control system in song birds as a model of neuronal plasticity (for review see Van der Linden et al., 2004), Tindemans et al. (2003) were able to show an activity dependent transsynaptic transport of Mn^2+^ from the site of local cerebral injection of Mn^2+^ in the HVC (high vocal center; a relay region of the song control system) to more downstream regions [such as the nucleus robustus arcopallialis (RA) and the striatal area X]. Using dynamic MEMRI, the authors reported that both regions showed a more rapid accumulation of Mn^2+^ in the stimulated birds. After about 10 h, this difference to non-stimulated birds vanished only for RA, but not for area X, suggesting a differential functional connectivity of the two regions in the song circuitry. Considering these previous reports and the results presented here, we conclude that, even in the case of systemic MnCl_2_ injection, axonal and transsynaptic transport of Mn^2+^ is modulated by the activity state of the neuronal pathway. Our results further suggest that reduced neuronal activity due to blockage of sensory inputs attenuates the transport of Mn^2+^ from its initial accumulation site, while continuous neuronal activity promotes the transport of Mn^2+^ between neurons.

### Ketamine and NMDA receptor blockage

We used ketamine in an anesthetic dose (138 mg/kg) (Buitrago et al., 2008) in order to block NMDA receptor-related neuronal activity (Anis et al., 1983) and, thus, to avoid further entrance of Mn^2+^ in neurons (Itoh et al., 2008), which might be caused by transportation of the animals from the vivarium to the scanning room and/or their fixation inside the scanner. We are aware of the fact that ketamine has complex and not fully understood mechanisms of action and might lead to unspecific effects which are unrelated to anesthesia such as hyperlocomotion (Hayase et al., 2006) or antidepressant-like effects in low doses (Kavalali and Monteggia, 2012). However, in the present study we can exclude that ketamine has affected MEMRI signal intensities by itself, due to: (i) MnCl_2_ treatment was given chronically for 8 days and finished at least 12 h before ketamine injection; (ii) there was only a short interval (~30 min) between ketamine administration and the scanning procedure; and (iii) the within-subject design used here (ketamine treatment before both first and second scans).

## Conclusion

Taken together, we provide evidence for neuronal activity-dependent accelerated transport of Mn^2+^ to projection terminals and across synapses. This observation allows for a more careful design of the experiments using systemic MnCl_2_ treatment. At the same time, it adds another layer of components to the interpretation of the results obtained by MEMRI.

## Author contributions

SA-C and CW designed the study; SA-C performed the experiments and wrote the first draft of the manuscript; MC analyzed the data. All the authors discussed the results and approved the final manuscript.

### Conflict of interest statement

The authors declare that the research was conducted in the absence of any commercial or financial relationships that could be construed as a potential conflict of interest.
